# Supplementary material for: Modeling glioblastoma heterogeneity as a dynamic network of cell states
Source: Mol Syst Biol. 2021 Sep 16;17(9):e10105. doi: 10.15252/msb.202010105 (PMC8444284; doi:10.15252/msb.202010105)
Supplement: Supplementary file 6 — Source Data for Figure 5 [file MSB-17-e10105-s004.zip › Figure5A_sourcedata/GSEA_3017/hallmarks_stateD.GseaPreranked.1621934542601/HALLMARK_MYOGENESIS.html]

Details for gene set HALLMARK\_MYOGENESIS[GSEA]

|  || Dataset | state23017 |
| Phenotype | NoPhenotypeAvailable |
| Upregulated in class | na\_neg |
| GeneSet | HALLMARK\_MYOGENESIS |
| Enrichment Score (ES) | -0.32643837 |
| Normalized Enrichment Score (NES) | -1.356439 |
| Nominal p-value | 0.13184358 |
| FDR q-value | 0.26578686 |
| FWER p-Value | 0.983 |
Table: GSEA Results Summary

  

Fig 1: Enrichment plot: HALLMARK\_MYOGENESIS      
 Profile of the Running ES Score & Positions of GeneSet Members on the Rank Ordered List

  

| PROBE | GENE SYMBOL | GENE\_TITLE | RANK IN GENE LIST | RANK METRIC SCORE | RUNNING ES | CORE ENRICHMENT || 1 | ITGB1 |  |  | 147 | 0.260 | -0.0914 | No |
| 2 | MEF2A |  |  | 163 | -0.251 | -0.0816 | No |
| 3 | TPM2 |  |  | 229 | -0.257 | -0.1098 | No |
| 4 | NAV2 |  |  | 243 | -0.259 | -0.0977 | No |
| 5 | ATP6AP1 |  |  | 264 | -0.260 | -0.0909 | No |
| 6 | FGF2 |  |  | 287 | -0.263 | -0.0854 | No |
| 7 | MEF2C |  |  | 337 | -0.267 | -0.1004 | No |
| 8 | PRNP |  |  | 344 | -0.267 | -0.0821 | No |
| 9 | IFRD1 |  |  | 469 | -0.282 | -0.1538 | No |
| 10 | CKB |  |  | 554 | -0.292 | -0.1938 | No |
| 11 | APP |  |  | 626 | -0.306 | -0.2225 | No |
| 12 | IGFBP7 |  |  | 733 | -0.326 | -0.2765 | No |
| 13 | NQO1 |  |  | 756 | -0.333 | -0.2650 | No |
| 14 | PKIA |  |  | 794 | -0.342 | -0.2643 | No |
| 15 | MYH9 |  |  | 820 | -0.350 | -0.2537 | No |
| 16 | DTNA |  |  | 857 | -0.359 | -0.2508 | No |
| 17 | PDE4DIP |  |  | 866 | -0.362 | -0.2259 | No |
| 18 | GNAO1 |  |  | 997 | -0.412 | -0.2911 | Yes |
| 19 | PLXNB2 |  |  | 1015 | -0.423 | -0.2680 | Yes |
| 20 | FHL1 |  |  | 1048 | -0.445 | -0.2545 | Yes |
| 21 | NCAM1 |  |  | 1049 | -0.445 | -0.2163 | Yes |
| 22 | SPARC |  |  | 1127 | -0.493 | -0.2336 | Yes |
| 23 | CNN3 |  |  | 1181 | -0.552 | -0.2272 | Yes |
| 24 | ITGB5 |  |  | 1205 | -0.590 | -0.1945 | Yes |
| 25 | CLU |  |  | 1207 | -0.592 | -0.1445 | Yes |
| 26 | FST |  |  | 1254 | -0.715 | -0.1188 | Yes |
| 27 | CRYAB |  |  | 1292 | -0.917 | -0.0688 | Yes |
| 28 | CDKN1A |  |  | 1306 | -1.046 | 0.0108 | Yes |
Table: GSEA details [plain text format]

  

Fig 2: HALLMARK\_MYOGENESIS: Random ES distribution      
 Gene set null distribution of ES for **HALLMARK\_MYOGENESIS**
